# “I didn't know anything, but I learned over time”: The process of nurses attaining autonomy in Intensive Care Units

**DOI:** 10.17533/udea.iee.v41n3e09

**Published:** 2023-10-26

**Authors:** Nestor Naranjo, Inna Florez, Edna Gómez

**Affiliations:** 2 Nurse, Doctor, Titular Professor. Email: iflorezt@unicartagena.edu.co Universidad de Cartagena Colombia iflorezt@unicartagena.edu.co; 3 Nurse, Doctor, Titular Professor. Email: egomezb@unicartagena.edu.co Universidad de Cartagena Colombia egomezb@unicartagena.edu.co; 4 Faculty of Nursing, University of Cartagena, Cartagena (Colombia) Universidad de Cartagena Faculty of Nursing University of Cartagena Cartagena Colombia

**Keywords:** professional autonomy, intensive care units, critical care cursing, autonomía profesional, unidad de cuidados intensivos, enfermería de cuidados críticos, autonomia profissional, unidades de terapia intensiva, enfermagem de cuidados críticos

## Abstract

**Objective.:**

Understand the social processes experienced by nursing professionals and the meanings underlying autonomy in adult Intensive Care Units in the city of Cartagena (Colombia).

**Methods.:**

A qualitative study with a grounded theory approach was conducted. Fifteen semi-structured interviews were carried out with nursing professionals, and the analysis was based on the coding technique proposed by Strauss & Corbin.

**Results.:**

Of the respondents, fourteen were female and one was male, with ages ranging from 23 to 57 years. Experience in intensive care units ranged from 1 to 28 years, and none had postgraduate studies. After thematic analysis, the central category was obtained from four categories: adaptation process, applicability of autonomy exercise, building autonomous competence, and limitations to the exercise of autonomy.

**Conclusion.:**

Nursing professionals achieve their autonomy through a social process, based on different stages of learning when facing the environment of the units. It is grounded in decision-making and the power to act freely. However, barriers continue to hinder it, including limitations imposed by institutions, protocol-based interventions, social status, and individual differences among professionals

## Resumen

**Objetivo.:**

Comprender el proceso social que viven los profesionales de enfermería y los significados que subyacen a la autonomía en las Unidades de Cuidados Intensivos adultos en la ciudad de Cartagena (Colombia).

**Métodos.:**

Estudio cualitativo con enfoque de la teoría fundamentada, se realizaron quince entrevistas semiestructuradas en profesionales de enfermería, el análisis se realizó basado en la técnica de codificación propuesta por Strauss y Corbin.

**Resultados.:**

Los entrevistados eran catorce de sexo femenino y uno de sexo masculino, con edades entre los 23 y 57 años, la experiencia en unidades de cuidados intensivos oscilo entre 1 y 28 años y ninguno conto con estudios de posgrados. Tras el análisis temático se obtuvo la categoría central “Cuando yo entré no sabía, con el tiempo lo aprendí” a partir de cuatro categorías: proceso de adaptación; aplicabilidad ejercicio de la autonomía; construyendo la competencia autónoma; y limitaciones para el ejercicio de la autonomía.

**Conclusión.:**

El profesional de enfermería logra su autonomía a través de un proceso social, basado en diferentes etapas de aprendizaje al enfrentarse al ambiente de las unidades, se sustenta en la toma de decisiones y el poder de hacer las cosas de forma libre. Sin embargo, existen barreras que la siguen obstaculizando, entre ellas las limitaciones dadas por las instituciones, intervenciones basadas en protocolos, el estatus social y la individualidad entre los profesionales.

## Resumo

**Objetivo.:**

Compreender o processo social vivenciado pelos profissionais de enfermagem e os significados subjacentes à autonomia nas Unidades de Terapia Intensiva adulto da cidade de Cartagena (Colômbia)

**Métodos.:**

Estudo qualitativo com abordagem da teoria fundamentada, foram realizadas quinze entrevistas semiestruturadas com profissionais de enfermagem, a análise foi realizada com base na técnica de codificação proposta por Strauss y Corbin.

**Resultados.:**

Os entrevistados foram quatorze mulheres e um homem, com idade entre 23 e 57 anos, a experiência em unidades de terapia intensiva variou entre 1 e 28 anos e nenhum possuía pós-graduação. Após a análise temática, obteve-se a categoria central “Quando entrei não sabia, aprendi com o tempo” a partir de quatro categorias: processo de adaptação; aplicabilidade exercício de autonomia; construção de competência autônoma; e limitações para o exercício da autonomia.

**Conclusão.:**

O profissional de enfermagem conquista sua autonomia por meio de um processo social, baseado em diferentes etapas de aprendizagem diante do ambiente das unidades, baseado na tomada de decisão e no poder de fazer livremente. No entanto, existem barreiras que continuam a dificultá-lo, incluindo limitações dadas pelas instituições, intervenções baseadas em protocolos, status social e individualidade entre os profissionais.

## Introduction

The precedents that have marked the evolutionary development of nursing are related to the subordination and linkage of practice to other professions, a fact that has, in turn, limited disciplinary strengthening, independent practice, decision-making, and control of their actions; characteristics that frame the autonomy of professional practice.^1^ While nursing is considered a relatively new profession, in its beginnings, it was classified as a trade, and its activity lacked a theoretical basis that supported it, factors that led it towards its professionalization process and the pursuit of autonomy; this not only involved seeking independent action for the generation of judgments and decision-making but also entailed authority and responsibility for controlling their actions, oriented towards care based on their knowledge that underpinned their work.^2^ This is how Florence Nightingale identified the need to base care practice on scientific principles and emphasized the need for nurses to make judgments about care and healthcare^3^, achieving the construction of its conditions and characteristics as an autonomous profession, the qualification of its work, positioning, and improving its social image.^4^ However, disparate development and evolution worldwide have led to a practice with limited autonomy, given by the professionalization process in each country, which is influenced by the development of technical, scientific, social, and cultural characteristics peculiar to each.^5^

In the Americas region, a similar situation is experienced to that of Asian countries, as is the case in Colombia, despite nursing having a legal framework that establishes and supports its autonomous practice.^6^ A loss of visibility and autonomy is evident in areas such as clinical care practice, due to factors such as social status, healthcare system, interdisciplinary relationships, job demands, institutional regulations, monotony, and workload. Similarly, we find that the specific and general competencies that professionals in these areas should possess, activities not inherent to professional practice, personal traits of each professional, demotivation, and individualism during working hours are other factors that hinder autonomous practice.^7^

Clinical areas like Intensive Care Units (ICU) have evolved rapidly since their inception in Colombia. They are considered complex, and the care of individuals at risk of vital and functional impairment is mediated by technological advancements. Therefore, nursing professionals need to possess knowledge of critical and vulnerable health situations of care recipients, specific and general competencies, and integrate personal and ethical values to perform autonomously. This involves decision-making, providing timely and high-quality care based on responsibility, skill, and leadership, which promotes empowerment and professional differentiation.^8^ The aim of the study was to understand the social process experienced by nursing professionals and the meanings underlying autonomy in adult intensive care units in the city of Cartagena.

## Methods

Qualitative Study with a Grounded Theory Approach: 15 out of 18 invited nurses from various adult Intensive Care Units (ICUs) in the city of Cartagena, Colombia, participated. Three of them declined to participate, while the rest remained involved throughout the study. Theoretical sampling guided data collection until reaching saturation of meaning units. Initially, a field visit was conducted to meet with the nurse coordinators who facilitated the recruitment of participants who met the inclusion criteria: nursing professionals with more than one year of experience working in ICUs.

Semi-structured face-to-face interviews were conducted in a single session by one of the researchers, who had no employment affiliation with the institutions or any prior relationship with the participants. The interviews had an average duration of thirty to sixty minutes and took place in a quiet, uninterrupted space chosen by the participants, either in their homes or institutional settings. The research's guiding question was, 'What has been your experience since the beginning of your career as a nurse in the Intensive Care Unit?' The interviews were recorded and transcribed after completion, and field notes were also taken. Manual analysis was employed based on the coding technique proposed by Strauss and Corbin.^9^ The process involved data fragmentation, conceptualization, coding, elaboration, and integration, enabling both descriptive and analytical analysis through the implementation of analysis tools such as interpretive frameworks, recording techniques, analytical memos, matrices, and conceptual diagrams.

For each transcribed interview, the researcher conducted microanalysis, allowing for a line-by-line reading and constant comparison, resulting in 203 data codes. These initial categories emerged through constant comparison, representing the phenomenon under investigation."

The conceptualizations were grouped into prominent concepts, along with their evoked properties, referred to as 'substantive codes' and 'in vivo codes,' enabling theoretical comparisons through analytical memos. Additionally, similarities and differences were sought between the attributes of categories defined by emotional responses and the dimensions defined by feelings towards the phenomenon. Once the information had been fragmented, axial coding was performed, and through the coding paradigm, four categories and their ten subcategories were defined, culminating in selective coding, which unified the categories into a central category. The study's rigor criteria were maintained in terms of credibility, auditability, and transferability, allowing for the portrayal of the phenomenon from human experiences as perceived by the study subjects, thus achieving real and/or true findings. In this regard, participants were provided with feedback on their narratives to validate the interpretations, and they expressed their agreement with the fidelity of the transcribed accounts of their lived experiences.

Furthermore, the study described and detailed the participant selection process, the participants' characteristics, and the context in which it was conducted.^10^ The project was approved by the Research Ethics Committee of the Faculty of Nursing at the University of Cartagena and included informed consent, voluntary participation, authorization to record interviews, confidentiality, and discretion in handling the information.

## Results

Fifteen interviews were conducted and analyzed with nurses, comprising 14 females and 1 male, ranging in age from 23 to 57 years old. Their experience in the ICU ranged from 1 to 28 years, and none of them had completed postgraduate studies. The participant descriptions are presented in Table 1.

**Table 1:** Description of the 15 participants in the study.

Code	Sex	Age	Years of Experience in ICU	Work Duration	Institution's Legal Entity	Type of Contract
ICUNurse01	Females	32	9	12	Private	Undefined
ICUNurse02	Females	49	28	7	Private	Undefined
ICUNurse03	Females	34	11	6	Private	Undefined
ICUNurse04	Females	34	7	2	Private	Service
ICUNurse05	Females	57	22	32	Private	Undefined
ICUNurse06	Females	44	22	6	Private	Fixed term
ICUNurse07	Females	31	8	6	Private	Fixed term
ICUNurse08	Male	26	1	1	Private	Fixed term
ICUNurse09	Females	28	6	6	Public	Fixed term
ICUNurse10	Females	44	17	3	Public	Fixed term
ICUNurse11	Females	37	15	4	Public	Fixed term
ICUNurse12	Females	52	18	9	Public	Fixed term
ICUNurse13	Females	47	20	9	Public	Fixed term
ICUNurse14	Females	23	1	1	Public	Fixed term
ICUNurse15	Females	43	15	6	Public	Fixed term

**Figure 1:**
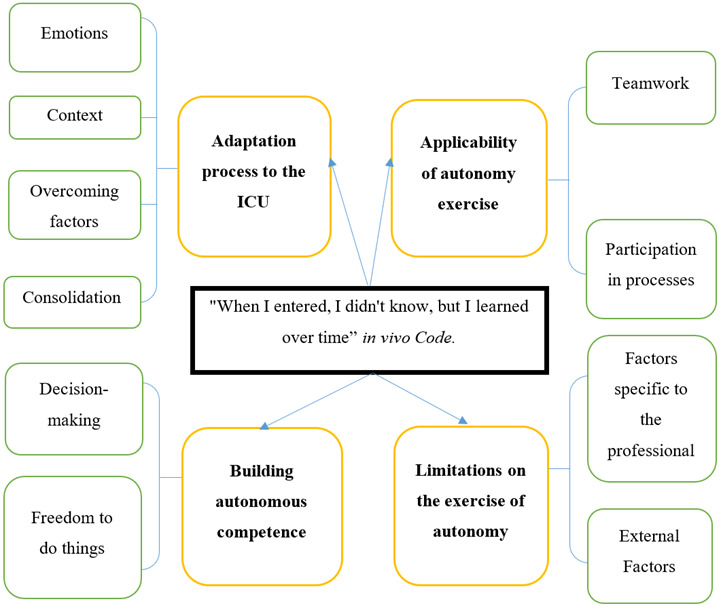
Central Category: 'When I entered, I didn't know, but I learned over time.' In vivo code.

Four categories and ten subcategories were merged, as represented in Figure 1.

### Category 1. Adaptation Process to the ICU

In the participants' discourse, it emerged that achieving professional autonomy requires an adaptive process to the ICU, involving different stages from their initial entry. Nurses exercise autonomy when they are confronted with situations that require leadership and the fulfillment of objectives supported by knowledge, leading them to develop their critical capacity to make decisions and projecting a problem-solving image during events. This, in turn, involves a process of learning, maturation, and dynamic social interaction with shared objectives among healthcare team professionals, enabling them to acquire, strengthen, and consolidate competencies, skills, and abilities.

*Emotions.* When professionals first face the ICU, they express emotions such as fear and uncertainty due to their inexperience, limited knowledge, and the skills required to perform and respond to the presented situations: "At first, well, some fear of the new, both medications that are not usual, handling them, the speed, infusions, and how to prepare them." (*ICUNurse12*).

*Context.* For professionals, ICUs are dynamic and complex environments due to the technology, high-risk medications, and the knowledge required for optimal performance, stemming from the care needs and demands of patients, their vulnerability, the complexity of functional impairments, and the risk to their lives: “It's a quite complex area, requiring a lot of attention, a lot of dedication because these are patients who need greater care, and in addition, there are many pieces of equipment that we may not use in other services.” (I*CUNurse04*).

*Overcoming Factors.* Nevertheless, despite experiencing fear and uncertainty, the professionals managed to confront and transform these emotions into something positive to acquire and strengthen their knowledge. They relied on self-learning processes, continuing education, interactions with other professionals, and colleagues with more experience. This encouraged the pursuit of knowledge and the acquisition of technical skills: “The most positive thing for us at the beginning was that training was provided, and there was also support, both administratively and from the coordinators, which helped alleviate our fears.” (*ICUNurse12*).

*Consolidation.* Over time, concurrently with the overcoming factors, they managed to understand, learn, strengthen, research, and consolidate the scientific foundations of care practice through interactions with other professionals or through their experiences. This was viewed as a social process that involved responding to the unknown and the challenges posed by the dynamics, technology, and medical management provided in ICUs: 'Knowledge is acquired through studying, interacting with others, with medical personnel, experimenting, and the day-to-day experience in the ICU. It's based on putting out fires and staying at the forefront of changes because things evolve, and one adapts to changes in infrastructure, technology, and changes in medical management. This is acquired through experience, reading, and self-improvement.' (*ICUNurse13*)."

### Category 2. Applicability of Exercising Autonomy

As nurses adapt to the ICU environment, they channel their efforts toward achieving and exercising professional autonomy by empowering themselves in two crucial aspects: 

*Teamwork.* ICU nurses emphasize that for them, it is more important to be a leader than a boss because they gain greater visibility in their practice through the strengthening of relationships, interaction with their team, and leadership. This positively strengthens professional empowerment and how they are perceived by the healthcare team. It also contributes to the acquisition of skills, knowledge, and expertise for the care of critically ill patients: “I take the leadership of the department, so I consider myself a leader in the department because I must ensure that everything is under control in the department, both with the doctors. Just the other day, a doctor said,” “If you don't function, the ICU doesn't function.” (*ICUNurse14*).

*Participation.* The participants agree that the exercise of autonomy is directed towards their active participation in processes that occur during patient care, such as medical rounds and critical patient situations. During these situations, they inquire and actively gather information about the patients, and nurses provide suggestions to guide care. They transition from being supervised to supervising care: “If the patient's condition worsens for any reason, it's our duty to inform the doctor or specialist so that they can take the necessary measures, and we ensure that all the patient's requirements from the physiotherapy service are met. In this aspect, we oversee that the patient receives what they need at that moment.” *(ICUNurse07).*

### Category 3. Building Autonomous Competence

Throughout the entire process experienced by nursing professionals in the ICUs, underlying meanings emerge, encompassing two subcategories: decision-making and the freedom to do things. These subcategories serve as a cross-cutting axis grounded in knowledge and expertise that transform them into experts and leaders in the ICU.

*Decision-Making.* The meaning attributed to autonomy by professionals’ centers around the freedom or power to make decisions once they feel prepared, free, and confident. However, some decisions are context-dependent, particularly in critical situations. These decisions must be well-founded since they cannot be made without a scientific basis to justify their rationale. They are characterized by being made freely, confidently, without consultation, assertively, in a timely manner, and of high quality, all with the goal of preserving patients' health: “It's the power to make your own decisions in certain moments, and you must have the knowledge to do so because you wouldn't make a decision without knowing” (*ICUNurse10*); “Well, I did it by always, as I told you, making the most appropriate decisions for the patients, for the unit. I did it with the confidence and the sense of belonging that characterizes me. Those were the two pillars that helped me exercise this autonomy.” (*ICUNurse08*).

In decision-making, professionals focus on seeking benefits related to three aspects: the multidisciplinary team, the unit, and the patient. Implicitly, the ethical principle of Beneficence/Non-Maleficence is evident, particularly in the care of the patient. Some decisions arise in critical circumstances that must be coordinated, validated, and supported by the multidisciplinary team. In this regard, nurses integrate knowledge from other disciplines to support care, demonstrating leadership, commitment, and ethical awareness: “I believe that to have autonomy, one must have knowledge. This knowledge must be based on scientific evidence through which we make decisions, at least in the field of healthcare. We make decisions to preserve the patient's health and provide holistic care.” (*ICUNurse07*).

In cases where decisions are solely directed by the nursing professional, the decisions and their justifications are based on the need for quick action due to the physiological change’s patients are experiencing to prevent complications that could lead to death. However, professionals validate and require legal support in the patient's medical record from the physician: “If a patient has hypoglycemia, I am autonomous and I know that I have to administer a dextrose bolus to the patient without needing to inform the doctor. That's what should be done, but it takes time; then I inform the doctor and say, Doctor, the patient had hypoglycemia, and I administered 200 of dextrose. Could you please prescribe it for me?" (*ICUNurse01*).

*Freedom to Act*. The other aspect underlying the meaning of autonomy is related to the freedom to act based on the ethical principles that support nursing. Autonomy is focused on activities and procedures specific to nursing care, such as catheterization, skin care, airway management, providing comfort and safety. These actions are supported by the skills acquired through practice and experience to execute them: “We are autonomous when it comes to placing devices and catheters. I don't have to wait for the doctor's order for that. In fact, we have the responsibility to ensure that the patient is well. I don't have to wait and call the doctor.” (*ICUNurse13*).

### Category 4. Limitations to the Exercise of Autonomy

As aspects that limit autonomy, barriers emerge as a cross-cutting axis in the process experienced by nurses, and these barriers are related to factors inherent to the nurses themselves and external factors:

*Factors Inherent to the Nurses Themselves.* These factors are focused on the personal characteristics of nurses and the nature of the nursing discipline. They encompass aspects such as personality, skills, and the ability to make decisions without prior discussion: “Sometimes they see it as being too strict, which is the way it should be, but at times, in an attempt to build camaraderie, one may lose the ability to lead. I believe that is also part of one's personality; I'm not naturally rigid.” (*ICUNurse03*).

*External Factors.* These factors are represented by the limitations imposed by institutions, interventions based on guidelines and protocols, social status, and the loss of fields of action that were once integrated with other disciplines. However, nursing care is not completely disconnected, but its autonomy is somewhat limited: “In Colombia, I see that they don't consider us an important profession; it's as if we were just anyone, like servants of the doctors. In contrast, in other places, nurses have a different status. In the United States, a nurse is an important person because people know that they are with the patient, and what they say matters because they are the ones with the patient all the time. Here, it's not like that.” (*ICUNurse06*).

**Figure 2:**
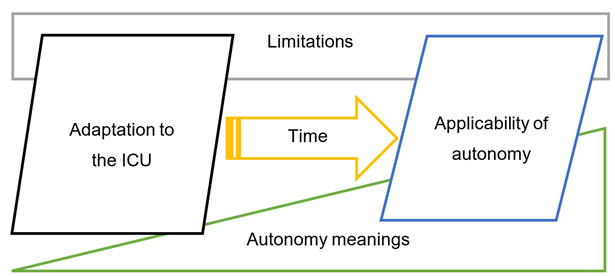
Adaptation Process and Applicability of Autonomy for Nursing Professionals in Intensive Care Units.

The graphic representation of the adaptation process and the applicability of autonomy is depicted in Figure 2.

## Discussion

The autonomous practice of nurses in ICUs involves an adaptive social process in which contextual, personal, and social factors related to the nursing discipline, the multidisciplinary team, and the unique characteristics of care required for critically ill patients come into play. It begins when they are first exposed to the units, mediated by emotional responses generated by the unfamiliarity of the context, and it ends when they become autonomous and empowered with knowledge, skills, and abilities to make decisions supported by self-learning processes. Gallego *et al*.^11^ point out that ICUs are specialized and hostile spaces that present urgent situations due to the type of patients being treated, evoking negative thoughts in professionals due to fear of what they are facing. Similarly, González^12^ states that nursing professionals acquire technical skills through continuous interaction with other disciplines, tangible problems that arise, and exchanges with experienced nurses, leading to gradual improvement and skill acquisition.

Likewise, professionals require knowledge that enables them to guide and provide a foundation for their practice, as well as demonstrate skills related to planning care interventions and their outcomes. In this regard, the applicability of autonomous practice occurs when nurses combine technical skills and scientific knowledge to exercise leadership and empower themselves as members of the ICU healthcare team through their active participation once they adapt. These findings are consistent with those presented by Jaimes.^13^ who states that autonomy is a condition that is not expected to emerge suddenly or be static, but rather that this condition is constructed based on various foundations and methodological factors, forging the path toward meeting the requirements that promote its applicability during professional practice. In line with this thinking, Tapp *et al*.^14^ suggest that achieving autonomy in nursing practice involves both a contextual and a personal interface, where autonomy develops over time, based on personal and professional growth. Similarly, Herrera *et al*.^15^ affirm that the development of nursing professionals is a continuous, articulated, and upward process for the acquisition of theoretical and practical knowledge. When integrated with disciplinary ethical norms and codes, this process enhances their professional being, doing, and performance, setting them apart from other healthcare disciplines and providing them with the necessary capacity to resolve situations in the exercise of professional autonomy. Similar results are reported by González.^12^, who found that ICU nursing professionals required constant integration of theory and practice, based on skills, personal initiative, the ability to make judgments, critical thinking, reflection, and the management of emotional aspects such as fear of the unknown, in order to achieve a learning process directed toward providing holistic and quality care. However, Marriner *et al*.^16^ in relation to Benner's postulates, note that disciplinary nursing knowledge is acquired and strengthened through nursing practice. This knowledge is acquired thanks to the various learning processes that nurses go through in different contexts, allowing them to transition from being inexperienced to becoming experts.

In the experience of ICU nurses, the underlying meaning of autonomy refers to the freedom to make decisions and the ability to do things without the supervision or authorization of other professionals. It is based on their experience and the technical-scientific skills that provide them with the confidence and assurance to carry out actions for the benefit of the patient, the unit, and the healthcare team. Gómez.^17^ points out that autonomy involves decision-making and independent practice based on the professional roles in different contexts in which nurses work. Furthermore, Muñoz *et al*.^18^ mention that one of the most important competencies for nursing professionals is related to decision-making, based on two aspects: personal attitudes and skills, and scientific knowledge and the complex processes underlying decision-making. This enhances the visibility of the professional in their problem-solving role and their self-confidence in observing, analyzing, and providing solutions based on their experience to the situations that arise.

The results of the process of achieving autonomy are similar to those reported in the study by Villagra *et al*.^19^ where participants found that autonomy is a methodological or systematic practice among nursing professionals in the units, based on knowledge, confidence, and the ability to make decisions. Similarly, Gallego *et al*.^11^ concluded that the autonomy of nursing professionals in ICUs is influenced by experience, the skills they acquire during their practice, and their interaction with other professionals, which is enhanced through scientific validation achieved through education and research. During the process, nurses mention that interaction with other professionals, participation in the healthcare team, and the ability to make decisions freely contribute to the autonomous practice of nursing in ICUs. These findings are consistent with the results of the study by Tapp *et al*.^14^ who found that autonomy is implicitly related to decision-making and nursing actions in various areas, including ICUs. They also found that the close relationships that nurses have with professionals allow them to create bonds characterized by camaraderie, based on respect and recognition.

The concept related to the freedom to do things independently without supervision or direction from another professional is central to nursing practice. According to Jaimes.^13^ autonomous practice in nursing involves activities focused on diagnosis, care, and treatment, within the nursing professional's role and scope of practice. These activities may involve collaboration with other healthcare professionals but highlight the nursing role in empowerment and the initiative of professionals to carry them out. Similarly, Gallego *et al*.^11^ concluded that contemporary nursing care has evolved to a higher level of expertise and competence, as it is now based on patient needs and prioritized, planned, and grounded in evidence.

The findings related to factors that continue to limit autonomy refer to characteristics inherent to nurses, such as lacking the necessary skills to exercise autonomy, and external factors related to the institutions where they work and their social status. Bonfante *et al*.^4^ suggest that one factor hindering autonomous practice is the individuality among nursing professionals. Gómez.^17^ notes in their study that while nursing professionals have disciplinary knowledge, they are still often influenced by the knowledge and practices of other professions, preventing full control of their practice. Additionally, Lopera *et al*.^7^ found that nurses lose their autonomy when their professional practice becomes burdened by activities that are not within the scope of nursing or when these activities are imposed by the institution. Similarly, Sánchez.^20^ mentions that a challenge in the nursing discipline and its field of practice is the social image projected by nurses, which often depicts them in secondary roles associated with other professions, lacking responsibility and autonomy in decision-making and having limited academic qualification.

The conclusion of this study is that the autonomous practice of ICU nurses underlies an adaptation process to the complex and dynamic environment in which it takes place, involving different stages to achieve decision-making, leadership, and participation in the multidisciplinary team. This social process enables nurses to guide their clinical practice and interventions with scientific, disciplinary, and ethical foundations. Gaining autonomy in the ICUs means for nurses making decisions and being able to do things freely, without the accompaniment, consultation, or direction of other professionals, based on technical-scientific disciplinary knowledge that provides them with confidence, security, and is supported by ethical/legal aspects related to the practice of nursing, while respecting the boundaries of other professionals. However, the social process reveals intrinsic and extrinsic factors such as individuality among professionals, lack of control in the practice of their profession, activities unrelated to care, and the guidelines of healthcare organizations that interfere with decision-making, factors that hinder the exercise of autonomy in clinical practice in ICUs.

Considering the idiographic nature of qualitative research, the generalization of the results to the entire population of nurses is considered a limitation. Consequently, the results can only be transferred to populations with the same characteristics as the study participants
